# A Microwave Differential Dielectric Sensor Based on Mode Splitting of Coupled Resonators

**DOI:** 10.3390/s24031020

**Published:** 2024-02-05

**Authors:** Ali M. Almuhlafi, Mohammed S. Alshaykh, Mansour Alajmi, Bassam Alshammari, Omar M. Ramahi

**Affiliations:** 1 Electrical Engineering Department, College of Engineering, King Saud University, Riyadh 11421, Saudi Arabia; malshaykh@ksu.edu.sa (M.S.A.); 439101302@student.ksu.edu.sa (M.A.); 439102104@student.ksu.edu.sa (B.A.); 2 Electrical and Computer Engineering Department, University of Waterloo, Waterloo, ON N2L 3G1, Canada

**Keywords:** avoided mode crossing, complementary split-ring resonator, microwave coupled resonators, microwave electric coupling, microwave near-field sensors, split-ring resonator

## Abstract

This study explores the viability of using the avoided mode crossing phenomenon in the microwave regime to design microwave differential sensors. While the design concept can be applied to any type of planar electrically small resonators, here, it is implemented on split-ring resonators (SRRs). We use two coupled synchronous SRRs loaded onto a two-port microstrip line system to demonstrate the avoided mode crossing by varying the distance between the split of the resonators to control the coupling strength. As the coupling becomes stronger, the split in the resonance frequencies of the system increases. Alternatively, by controlling the strength of the coupling by materials under test (MUTs), we utilize the system as a microwave differential sensor. First, the avoided mode crossing is theoretically investigated using the classical microwave coupled resonator techniques. Then, the system is designed and simulated using a 3D full-wave numerical simulation. To validate the concept, a two-port microstrip line, which is magnetically coupled to two synchronous SRRs, is utilized as a sensor, where the inter-resonator coupling is chosen to be electric coupling controlled by the dielectric constant of MUTs. For the experimental validation, the sensor was fabricated using printed circuit board technology. Two solid slabs with dielectric constants of 2.33 and 9.2 were employed to demonstrate the potential of the system as a novel differential microwave sensor.

## 1. Introduction

In 1952, Schelkunoff et al. proposed a method for increasing the permeability of artificial dielectrics by introducing a loop loaded with capacitance, effectively a loop-split resonator [1]. This led to the discovery that magnetic polarizability experiences resonance when approaching a frequency related to the capacitance and inductance of the loop [1]. In 1999, Pendry et al. introduced new engineered materials (metamaterials) using electrically small resonators called SRRs [2]. Later, the complements of SRRs (CSRRs) were introduced [3]. The introduction of SRRs was a significant development in electromagnetic research, leading to new applications that were never possible before. To name a few examples, metamaterials have been adopted in many technologies, such as filters [4,5,6], mutual coupling mitigation [7], antennas [7,8,9,10,11], and noninvasive glucose detection [12]. Furthermore, microwave planar electrically small resonators, such as SRRs and CSRRs, have been widely utilized in various applications. At resonance frequencies, highly concentrated electromagnetic energy is confined within small regions [2], making these resonators ideal for use in designing planar microwave sensors. Indeed, several sensing modalities have been proposed and reported [13,14,15,16,17,18,19]. These sensors are sensitive, compact, reusable, and inexpensive. With the development of low-loss substrates and printed circuit board technology, significant progress has been made toward printing different types of microwave planar sensors.

In general, the sensing mechanism is based on measuring resonance frequency shifts relative to a reference point, such as air or free space. Compared with CSRR-based sensors, SRR-based sensors have smaller sensing areas, making them more suitable for compact applications [20,21,22,23]. However, CSRR-based sensors are more sensitive, which can be attributed to the larger sensing areas of their resonators [24]. Nevertheless, various techniques have been proposed to enhance the sensitivity for these resonators [14,15,19,25,26,27].

Microwave multiresonator sensors for differential and comparison sensing have been reported in several works, including [28,29,30,31]. For example, to enable differential sensing, microstrip lines containing a pair of identical resonators (such as stepped impedance resonators (SIRs), CSRRs, and SRRs reported in [28,30,31]) were utilized. One resonator was loaded with a well-characterized material (reference sample), while the other resonator was loaded with the MUT, resulting in the appearance of two notches in the sensor’s spectrum, which represents the dis-similarity between the samples. In those studies, the resonators were sufficiently separated in order to prevent any coupling between them, as the coupling was regarded as a degradation to the sensitivity [28,30,31]. Nevertheless, the two resonance frequencies (two notches) depend not only on the resonators but also on the length of the splitter/combiner parts [28,30,31]. It is worth emphasizing that the frequency splitting is not based on inter-resonator coupling; it is fundamentally based on breaking the symmetry by loading one of the uncoupled resonators with the MUT. Thus, inter-resonator coupling remains undesirable and must be completely eliminated or reduced as much as possible for differential sensing.

Furthermore, in [32], three nonidentical rectangular resonators were utilized to have three distinguishable resonance frequencies that are essential for measurement, allowing for simultaneously characterizing many parameters. Thus, multiresonator sensors can offer many advantages over a single resonance sensor. It has been stated that the precision of the resonators’ design requires having three nonidentical resonance frequencies. In addition, a three-identical CSRR sensor integrated with artificial intelligence was reported in [33].

Despite the widespread use of planar resonators in microwave sensing applications, the idea of employing coupled resonators to further improve the sensitivity and selectivity of these sensors has only recently gained attention. Thus far, only a few studies have investigated the use of multiple-coupled-CSRR-based sensors to enhance sensitivity. In [34], coupled CSRRs were aligned along an axis perpendicular to the direction of propagation. Thus, by loading the sensing area with the MUT, the resonance frequencies of the individual resonator are altered, as well as the mutual coupling’s capacitance. While coupled CSRR sensors show high sensitivity, the sensing areas of the sensors become larger compared with a single CSRR. However, in [34], the sensing mechanism was based on the traditional method of monitoring the shift of a single resonance frequency.

In this paper, we expand the concept of coupled resonators to demonstrate the avoided mode crossing in SRRs. Avoided mode crossing is a phenomenon that can be observed in any coupled cavity system, characterized by mode splitting in the transmission scan of the cavity [35]. It is a well-established phenomenon in the optical regime that has been observed in optical atomic systems and optical microcavities [10,36,37,38]. More generally, this effect can be observed in any coupled cavity system (e.g., superconducting resonators [39,40]). In this work, we utilize the resulting mode splitting of the resonance frequencies as a differential sensing mechanism. The proposed sensor is based on a differential sensing setup that can be applied for a variety of applications.

The paper is organized as follows: First, we introduce the concept of avoided mode crossing and develop the theoretical background using classical techniques for microwave-coupled resonators. Next, we investigate avoided mode crossing by varying the distance between two coupled SRRs, thereby controlling the electric coupling and the observed split in the resonance frequency. The system is designed and simulated using a 3D numerical simulation from Ansys HFSS [41]. We study the effects of using both eigenmodes and full wave solvers to analyze the scattering matrix. By carefully designing the microwave electric coupling (EC) between the resonators and placing dielectric MUTs between them, we demonstrate that the mode split becomes dependent on the MUT dielectric constant. This controlled split can be beneficial for designing novel differential microwave sensors. A sensing system based on a two-port microstrip line used to excite two coupled synchronous SRRs was fabricated using printed circuit board technology. Finally, we conduct a proof-of-principle experiment employing two dielectric slabs with dielectric constants of 2.33 and 9.2 to demonstrate the potential of the proposed differential sensor.

## 2. Avoided Mode Crossing at Microwave Regime: Theory

**Figure 1:**
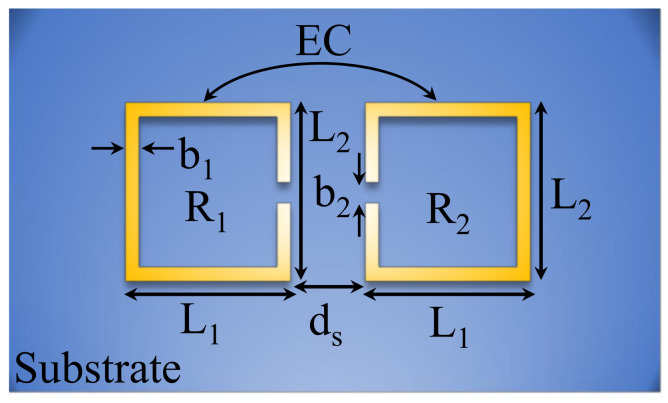
Schematic of two coupled synchronous SRRs where the inter-resonator coupling is based on electric coupling.

Consider two coupled synchronous SRRs denoted as $R_{1}$ and $R_{2}$, with a separation distance $d_{s}$, as shown in Figure 1. These resonators are coupled to an external circuit through magnetic coupling, specifically a 50 Ω two-port microstrip line. While this study specifically used SRRs, the underlying concept and methodology can be applied to other types of resonators as well. For the demonstration of the theory, the inter-resonator coupling is chosen to be microwave electric coupling, since the concept of avoided mode crossing will be demonstrated by designing a dielectric sensor. However, other types of coupling can also be considered [42]. For this section, we will focus on the coupled resonators excluding the external coupling circuit in the analysis.

Coupled resonators can be of any type of structure and have their self-resonance frequencies. The general electric and magnetic coupling coefficient (κ) for two coupled resonators can be expressed using the theory developed in [43] as

(1) $$\kappa =\kappa_{E}+\kappa_{M}.$$

Since electric coupling is predominant in the structure shown in Figure 1, the magnetic coupling can be ignored. In this case, the electric coupling coefficient ($\kappa_{E}$) can be expressed as [43]

(2) $$\kappa_{E}=\frac{\;\int \int \int \;\varepsilon E_{1}\cdot E_{2}dv}{\sqrt{\;\int \int \int \;\varepsilon {\left|E_{1}\right|}^{2}dv\times \;\int \int \int \;\varepsilon {\left|E_{2}\right|}^{2}dv}},$$

where $E_{1}$ and $E_{2}$ represent the electric field of each resonator at the resonance frequency [42]. The dot product between the fields can produce a positive or negative sign, indicating that the electric can either support or cancel each other out [42], and ϵ represents the permittivity of the entire effective volume that contains the electric field components. Therefore, by disrupting the electric field distribution with a dielectric material under test, changes in $\kappa_{E}$ can be observed. This observation forms the basis of the designed sensing system.

**Figure 2:**
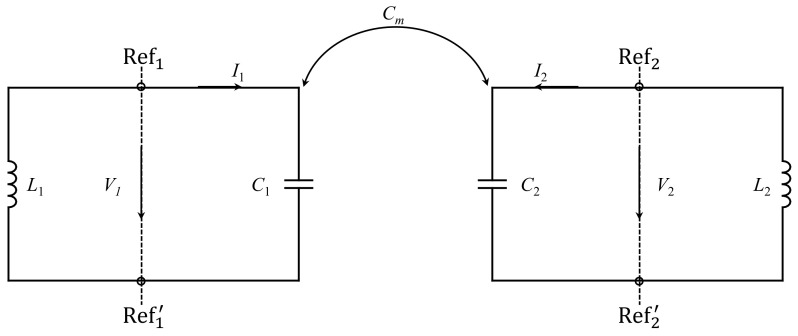
The circuit diagram of the coupled SRR shown in Figure 1.

Figure 2 shows the lumped-circuit model used to analyze the coupling between the resonators in Figure 1 and to predict the avoided mode crossing [42]. In the model, $C_{m}$ represents mutual capacitance, while $C_{1}$, $L_{1}$, $C_{2}$, and $L_{2}$ represent the inductance and capacitance of the resonators. Since SRRs in this study are synchronous (identical), $C_{1}$ = $C_{2}$ and $L_{1}$ = $L_{2}$. The circuit model is only valid near the resonance frequency. Based on the reference planes ${\mathrm{Ref}}_{1}-{\mathrm{Ref}}_{1}^{\prime }$ and ${\mathrm{Ref}}_{2}-{\mathrm{Ref}}_{2}^{\prime }$ and an equivalent circuit model (it is not shown here; readers can refer to [42]), electric and magnetic resonances can be expressed as

(3) $$f_{e}=\frac{1}{2\pi \sqrt{L\left(C+C_{m}\right)}}$$

(4) $$f_{m}=\frac{1}{2\pi \sqrt{L\left(C-C_{m}\right)}}.$$

Compared with the resonance frequency of an uncoupled resonator, which can be written as

(5) $$f_{u}=\frac{1}{2\pi \sqrt{LC}}.$$

Note that the electric resonance $f_{e}$ is lower than $f_{m}$, as the total capacitance increases with $C_{m}$, whereas $f_{m}$ is larger than the resonance frequency of the uncoupled resonator.

Using (3) and (4), the electric coupling coefficient ($\kappa_{E}$) can be written as [42]

(6) $$\kappa_{E}=\frac{f_{m}^{2}-f_{e}^{2}}{f_{m}^{2}+f_{e}^{2}}=\frac{C_{m}}{C}.$$

From (6), it can be observed that $\kappa_{E}$ is directly proportional to $C_{m}$ and inversely proportional to *C*. The contribution to C can be broken into two parts, the gap/split capacitance and the capacitance between the resonator and the ground plane. While it is expected that the mutual capacitance will depend on the gap capacitance, the expression suggests that reducing the transmission line capacitance can lead to higher coupling and splitting.

Interestingly, this problem has been addressed from a different point of view. Using the well-known coupled-mode theory [44], it can be shown that the resonance splits to

(7) $$\omega_{\pm }=\frac{\omega_{1}+\omega_{2}}{2}\pm \sqrt{{\left(\frac{\omega_{1}-\omega_{2}}{2}\right)}^{2}+{\left|K\right|}^{2}}.$$

For the synchronous resonator case ($\omega_{1}=\omega_{2}=\omega_{u}$), the coupling parameter |K| can be related to the electric coupling coefficient by substituting Equation (7) into Equation (6). Hence, the frequency split, Δf, can be written as

(8) $$\Delta f=2|K|=\frac{2f_{u}-2f_{u}\sqrt{1-\kappa_{E}^{2}}}{\kappa_{E}}\approx \kappa_{E}f_{u},$$

where the approximation is valid for weak coupling. This simple expression provides a direct link between the magnitude of the split in resonance frequency and coupling (or the dielectric constant of the MUT, as will be discussed below). The validity of this expression will be analyzed in the next section by comparing it with an eigenmode solver.

## 3. Case Study: A Sensor Based on Two Synchronous Split-Ring Resonators

### 3.1. System Design

**Figure 3:**
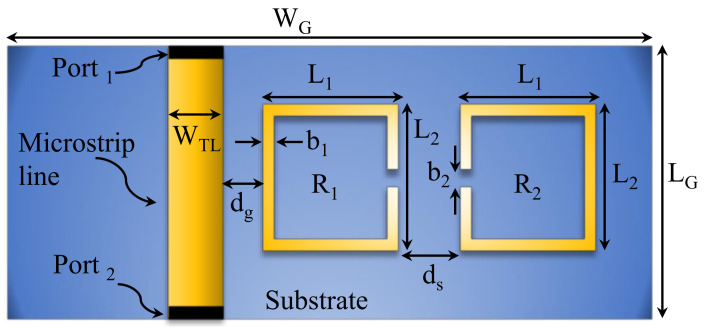
Schematic of two synchronous SRRs coupled to a two-port microstrip line (TL).

Figure 3 shows a two-port system based on a microstrip line (TL) that is utilized to excite two synchronous SRRs. The TL is designed on a substrate from Rogers Corporation (RO4350) with a dielectric constant of 3.66, a loss tangent of 0.0031, and a thickness of 0.73 mm. For a 50 Ω characteristic impedance, the calculated width of TL ($W_{\mathrm{TL}}$) is 1.56 mm. Note that, in principle, other substrates can be used. However, the RO4350 substrate is available commercially, and compared with the FR-4 substrate, it offers lower losses at the expense of a higher cost. The electric coupling ($\kappa_{E}$) will be numerically investigated by varying $d_{s}$. For the side length of SRR, $L_{1}$ and $L_{2}$ are chosen to be 4 and 11 mm, respectively. These particular values are optimized to increase the coupling between TL and the resonator ($R_{1}$) such that the $|S_{21}|$ dip at the resonance frequency is minimized (i.e., approaching critical coupling). In addition, the dimensions are chosen to keep the operating frequencies below 9 GHz in order to measure the response using our vector network analyzer (N9925A 9 GHz FieldFox). The final design specifications are summarized in Table 1.

### 3.2. Numerical Simulation: Eigenmode-Solver-Based Analysis

To analyze the resonance frequencies ($f_{e}$ and $f_{m}$), the Ansys HFSS eigenmode solver was utilized [41]. The main advantage of using this solver is that it is faster and can easily find the first two modes. To use the solver correctly, a cavity with walls made of perfect electric conductor material (PEC) was initially designed to have the first mode that exceeds the resonance frequency of the intended system. This cavity was designed to be electrically larger than the intended system, so it would not impact the resonance frequencies and provide an accurate prediction. It is recommended to design the cavity using (9) [45], and then simulate the cavity using the solver to extract the modes. The first mode should be greater than the expected resonance frequencies of the system. Note that the microstrip line was not included during the analysis. The following bullets summarize the steps followed for the eigenmode-solver-based analysis:

1. In the 3D simulation (HFSS), choose the solution type to be eigenmode;
2. Design a rectangular metallic cavity where the resonance frequency of the dominant mode must be greater than the expected resonance frequencies of the resonators;
3. Make the boundary of the cavity with walls made of perfect electric conductor material (PEC);
4. In the eigen solution step, choose the minimum frequency to be smaller than the first resonance frequency of the intended system;
5. In our case, as we are interested in extracting the resonance frequencies ($f_{e}$ and $f_{m}$), choose the number of modes to be 2.

The rectangular metallic dimensions are width = 30 mm, length = 22.5 mm, and height = 25 mm. Using the following equation [45],

(9) $$f_{mnp}=\frac{u^{\prime }}{2}\sqrt{{\left[\frac{m}{a}\right]}^{2}+{\left[\frac{n}{b}\right]}^{2}+{\left[\frac{p}{c}\right]}^{2}},$$

where $u^{\prime }=\frac{1}{\sqrt{\mu \epsilon }}$, the calculated resonance frequency of the dominant mode ($TE_{101}$) is 7.81 GHz. Thus, the intended system’s resonance frequency must be lower than 7.81 GHz for an accurate prediction of the resonance frequencies, $f_{e}$ and $f_{m}$, and $f_{u}$. For all calculations in this section, the dimensions of the system without TL are exactly the same as those presented in Table 1, except for $L_{G}$ = 15 mm and $W_{G}$ = 20 mm.

**Figure 4:**
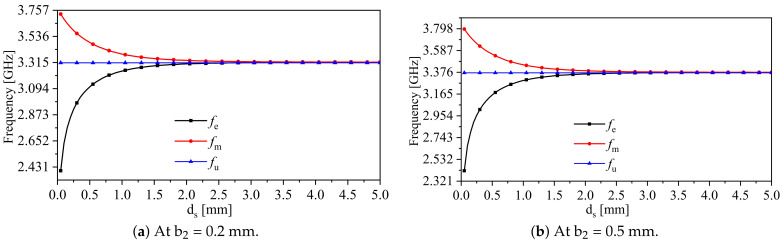
The system eigenmodes, $f_{e}$ and $f_{m}$, and $f_{u}$, versus the variable, $d_{s}$.

For calculating the resonance frequency ($f_{u}$) of a single SRR, the resonator was placed inside the metallic cavity. The resonance frequency was determined for two scenarios: $b_{2}$ values of 0.2 and 0.5 mm, resulting in resonance frequencies of 3.312 and 3.37 GHz, respectively. It can be observed that the capacitance *C* in (6) is inversely proportional to $b_{2}$. Therefore, the coupling factor ($\kappa_{E}$) is anticipated to be enhanced in the case of $b_{2}$ = 0.5 mm compared with $b_{2}$ = 0.2 mm. Furthermore, the coupling factor was examined by varying the parameter $d_{s}$ (the spacing between the resonators) from 0.05 to 5 mm, with a step value of 0.05 mm. As per Equation (6), it can be observed that $\kappa_{E}$ is inversely proportional to $d_{s}$ since the mutual capacitance ($C_{m}$) is, in fact, inversely proportional to $d_{s}$. The resonance frequencies, $f_{e}$ and $f_{m}$, and $f_{u}$, versus the variable $d_{s}$, with $b_{2}$ values of 0.2 and 0.5 mm, are plotted in Figure 4a,b, respectively.

**Figure 5:**
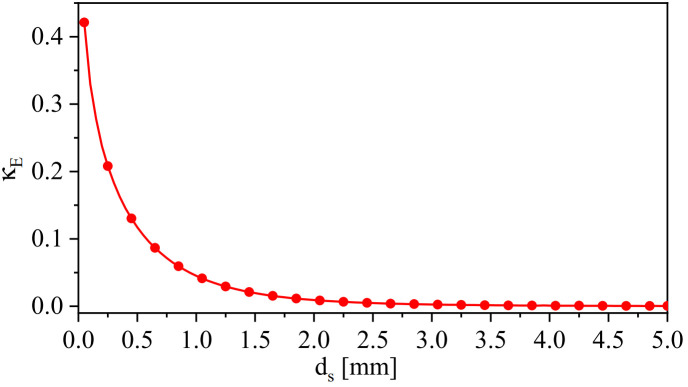
The electric coupling ($\kappa_{E}$) versus the variable, $d_{s}$, at $b_{2}$ = 0.5 mm, where $d_{s}$ is varied from 0.05 to 5 mm with a step value of 0.05 mm (Some values of $\kappa_{E}$ versus $d_{s}$ are denoted by red dots).

**Figure 6:**
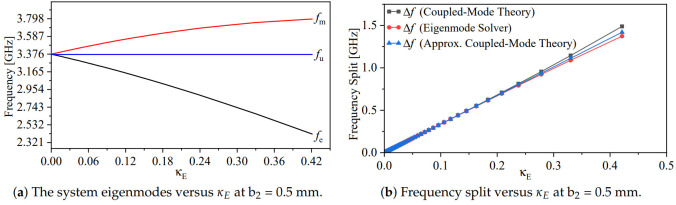
The frequency split of the system versus $\kappa_{E}$. (**a**) The red line represents the magnetic resonance ($f_{m}$) versus $\kappa_{E}$, the blue line represents the resonance frequency of a single resonator ($f_{u}$) versus $\kappa_{E}$, and the black line represents the electric resonance ($f_{e}$) versus $\kappa_{E}$ (**b**) The quantified frequency splitting versus $\kappa_{E}$ by coupled-mode theory using (8) in the exact (black line), weak coupling approximation (blue line), and the eigenmode solver in HFSS (red line).

As expected from Section 2, the coupled-synchronous-SRR-based system will produce two resonance frequencies, $f_{e}$ and $f_{m}$. The stronger the coupling (represented by smaller spacing min$\{d_{s}\}$) is, the stronger the split between the two resonance frequencies is. The electric coupling ($\kappa_{E}$) in terms of the variable $d_{s}$ can be calculated using (6). Figure 5 shows $\kappa_{E}$ versus $d_{s}$ when $b_{2}$ = 0.5 mm. It is evident that the electric coupling decays exponentially as the separation $d_{s}$ increases. By plotting the system eigenmodes, $f_{e}$ and $f_{m}$, versus the electric coupling ($\kappa_{E}$), one can easily observe that the split in the modes increases as $\kappa_{E}$ increases, as shown in Figure 6a. As expected from (6), at $b_{2}$ = 0.5 mm (smaller resonator’s capacitance) and $d_{s}$ = 0.05 mm, $\kappa_{E}$ is enhanced by almost 2%. Thus, $b_{2}$ can be optimized for higher coupling. Furthermore, the frequency split was quantified by coupled-mode theory using (8) in the exact and approximated form (weak coupling approximation) and then compared with the eigenmode solver in HFSS. The frequency split versus $\kappa_{E}$ is shown in Figure 6b. As expected, the theory gives an accurate prediction compared with the eigenmode solver at the weaker coupling.

### 3.3. Numerical Simulation: Scattering-Parameter-Based Analysis

**Figure 7:**
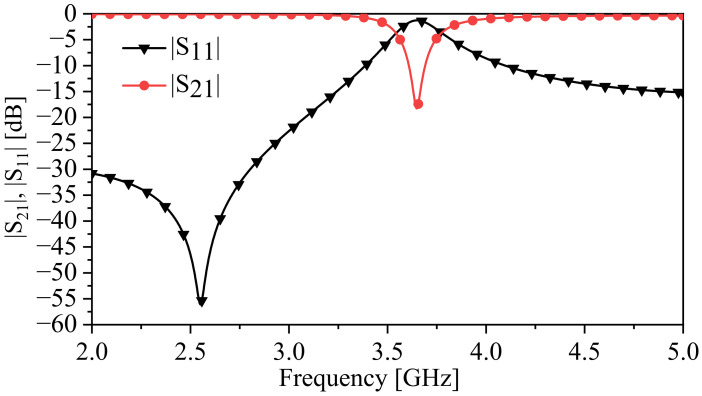
The response of the one-SRR-based system in the form of the transmission and reflection coefficients ($|S_{21}|$ and $|S_{11}|$) at $b_{2}$ = 0.5 mm.

The scattering-parameter-based analysis is a beneficial tool for designers to analyze the proposed system, either in the case of the unavailability of an eigenmode solver or for including the external circuit that is utilized to couple to the system. In addition, the final design of the proposed system will be fabricated and tested using a vector network analyzer, where the response of the system in the form of the scattering transmission coefficient ($|S_{21}|$) will be measured. We started our analysis by stimulating the one-SRR-based system to observe the response and to extract the resonance frequency, fu, as expressed in (5). Figure 7 shows the transmission coefficient ($|S_{21}|$). At the minimum transmission coefficient (min$\left\{|S_{21}|\right\}$), the resonance frequency is 3.65 GHz and the resonance quality factor is ≃13.44.

**Figure 8:**
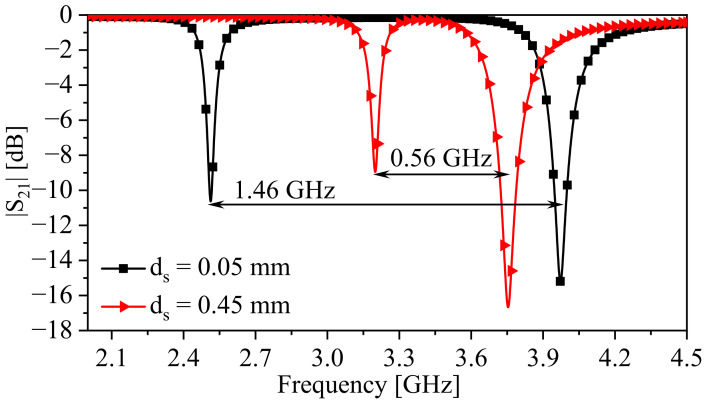
The response of the two-synchronous-SRR-based system in the case of $d_{s}$ = 0.05 and 0.45 mm at $b_{2}$ = 0.5 mm.

**Figure 9:**
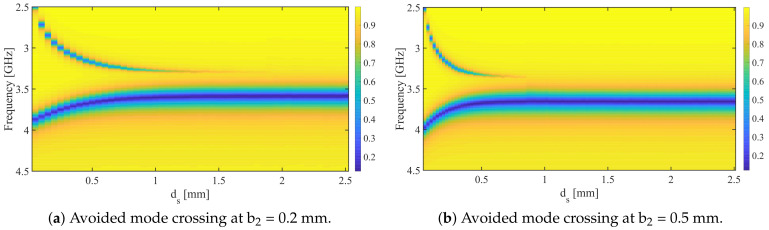
The transmission coefficient ($|S_{21}|$) in a 2D plane as a function of frequency and $d_{s}$.

Next, the two-synchronous-SRR-based system will be simulated, with the design specifications shown in Table 1, where $b_{2}$ is chosen to be 0.5 mm. The space between the resonators ($d_{s}$) is variable, ranging from 0.05 to 5 mm with a step value of 0.05 mm. Figure 8 illustrates the response of the system at two values of $d_{s}$, 0.05 and 0.45 mm. For $d_{s}$ = 0.05 mm, the resonance frequencies, $f_{e}$ and $f_{m}$, at min$\left\{|S_{21}|\right\}$, are 2.513 and 3.973 GHz, respectively, whereas in the case of $d_{s}$ = 0.45 mm, $f_{e}$ and $f_{m}$ are 3.2 and 3.754 GHz, respectively. It is evident that the split between $f_{e}$ and $f_{m}$ is inversely proportional to $d_{s}$. Furthermore, avoided mode crossing can be investigated by plotting the transmission coefficient ($|S_{21}|$) in a 2D plane as a function of frequency and $d_{s}$, as illustrated in Figure 9, for two chosen values of $b_{2}$ (0.2 and 0.5 mm). As predicted by the eigenmode-solver-based analysis, avoided mode crossing can be observed, and it is strong at smaller values of $d_{s}$, as a result of strong electric coupling between the resonators. This coupling can be disturbed, for example, by dielectric materials placed between the resonators if one wants to utilize the system as a sensor.

### 3.4. The Proposed System as a Microwave Differential Dielectric Sensor

**Figure 10:**
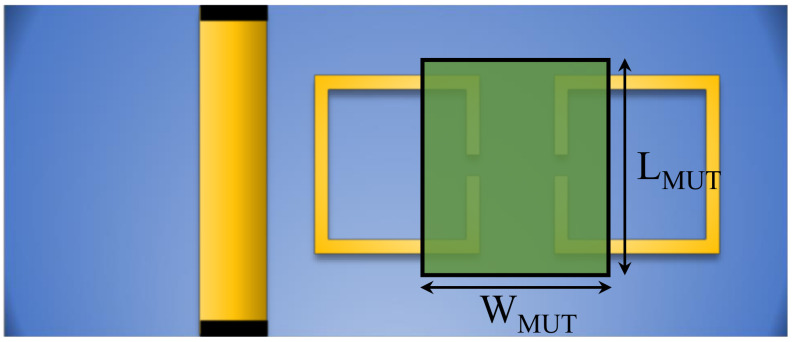
Schematic of two synchronous coupled SRRs loaded with a dielectric slab ($W_{\mathrm{MUT}}$ = 3.2 mm, $L_{\mathrm{MUT}}$ = 13 mm, and $T_{\mathrm{MUT}}$ = 3 mm).

**Figure 11:**
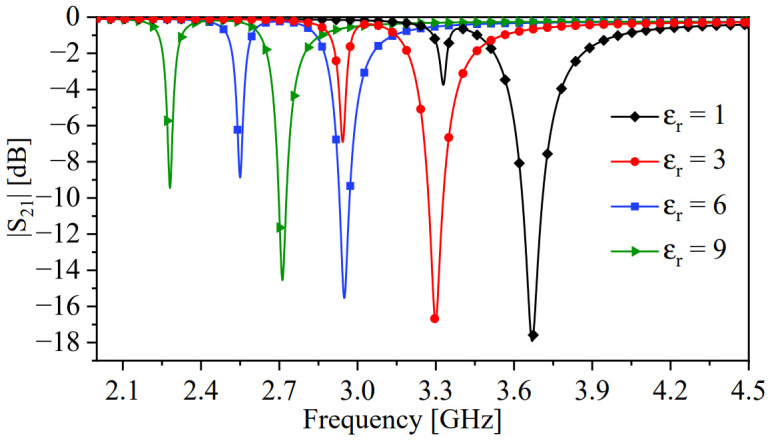
The transmission coefficient ($|S_{21}|$) of the system in the presence of a dielectric slab with a relative permittivities of 1, 3, 6, and 9.

The predominant coupling in the proposed system is the electric coupling, which can be disturbed or controlled by MUTs. Thus, the system can be utilized as a microwave sensor, as illustrated in Figure 10. To emphasize the effects of an MUT on mode splitting, we set the spacing between the resonators at the onset of mode splitting, which corresponds to a spacing (ds) of 1 mm. This choice is based on the numerical results shown in Figure 9. In addition, the spacing is chosen based on an MUT, with dielectric constants of 2.33 and 9.2, which will be utilized to demonstrate the effects of the proposed sensors. Note that the spacing between the resonators can be optimized based on the range of the intended dielectric constants to be tested. For instance, for high-dielectric materials, a larger separation would be better suited. A dielectric slab with a width ($W_{\mathrm{MUT}}$ = 3.2 mm), length ($L_{\mathrm{MUT}}$ = 13 mm), and thickness ($T_{\mathrm{MUT}}$ = 3 mm) is placed between the resonators, as shown in Figure 10. We first performed the numerical simulation by sweeping the dielectric slab’s constant from 1 to 11 with a step value of 0.5. Since the system’s response will overlap at different values of the dielectric constant, only certain values were selected to plot the response, as shown in Figure 11.

**Figure 12:**
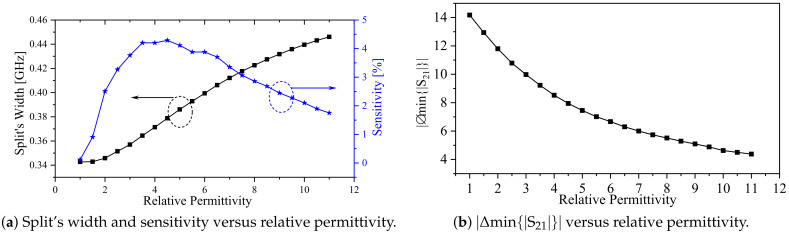
The system response versus the relative permittivity of the slab. (**a**) The blue and black lines represent the sensitivity and the degree of the frequency splitting of the system, respectively, in the presence of MUT. (**b**) The black line represents the absolute value of the difference in the magnitude min$\left\{|S_{21}|\right\}$ between the split resonances in the presence of MUT.

The effects of MUTs on the proposed system can be observed as shifts in the resonance frequencies $f_{e}$ and $f_{m}$, changes in magnitude at min$\left\{|S_{21}|\right\}$, and changes in the split’s width between $f_{e}$ and $f_{m}$. Figure 12a shows the changes in the split’s width as a function of the relative permittivity. Within the simulated values, the frequency splitting increases monotonically with the dielectric constant. The changes are relatively small, as anticipated from (6) as loading the system with an MUT will affect both $C_{m}$ and *C* simultaneously. However, our work establishes the theoretical foundation for linking the capacitance values ($C_{m}$ and *C*) to the width of the resonance split, which can be further explored in future studies using different resonator topologies. Additionally, in Figure 12b, the absolute value of the difference in the magnitude min$\left\{|S_{21}|\right\}$ between the split resonances is plotted against the relative permittivity, illustrating more prominent effects. Therefore, the combination of changes in the resonance split and the magnitude value of $\left\{|S_{21}|\right\}$ can be employed as a differential sensor to detect the presence of MUTs.

To quantify the performance of the sensor, we define the sensitivity in terms of the frequency split as

(10) $$S\equiv \frac{1}{\Delta f_{o}}\frac{\partial \Delta f}{\partial \epsilon_{r}},$$

where S, Δf, and $\Delta f_{o}$ are the sensitivity, the amount of frequency split, and the initial frequency split in air (i.e., $\epsilon_{r}=1$), respectively. The sensitivity curve is shown in Figure 12a in blue. Initially, the sensitivity is low but rapidly increases to 4.3% at a relative permittivity of 4.5. Afterwards, the sensitivity decreases, reaching 1.5% at a relative permittivity of 11. It is interesting to note that the location of maximum sensitivity is dependent on the spacing between the coupled resonators, which may be optimized for a specific targeted permittivity range.

## 4. Fabrication and Experimental Results

**Figure 13:**
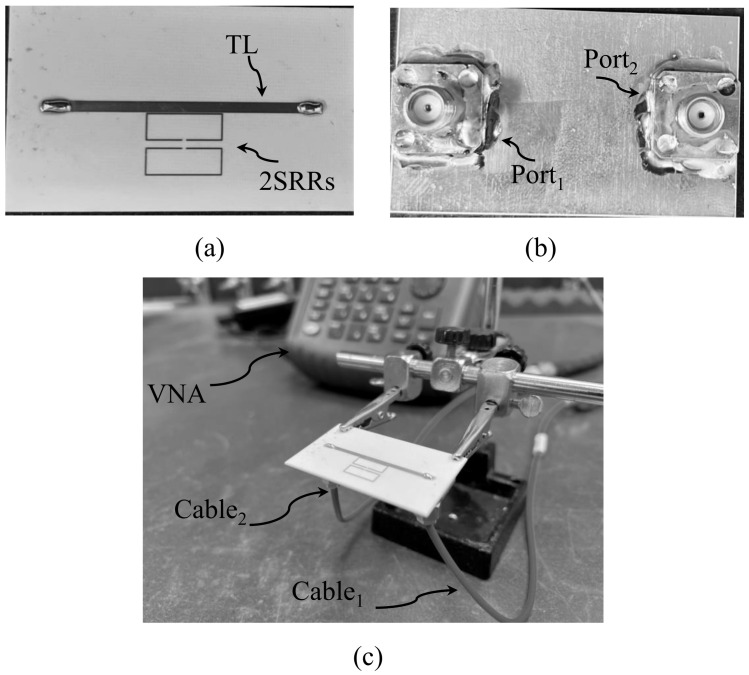
The fabricated two-port system: (**a**) top view, (**b**) bottom view, and (**c**) perspective view where the system is connected to a VNA.

The viability of the proposed technique was experimentally verified by fabricating a two-port microstrip line loaded with two synchronous coupled SRRs with dimensions presented in Table 1. The final design specifications for $d_{s}$ and $b_{2}$ are 1 and 0.5 mm, respectively. Figure 13 presents the fabricated system using PCB technology. To assess the performance of the fabricated sensor, it is necessary to measure the scattering parameters, which characterize the behavior of the system. The standard procedure for experimentally measuring the scattering parameters is to use a calibrated vector network analyzer (VNA). The VNA allows us to measure the complex reflection and transmission coefficients of the sensor, providing valuable information about its performance.

**Figure 14:**
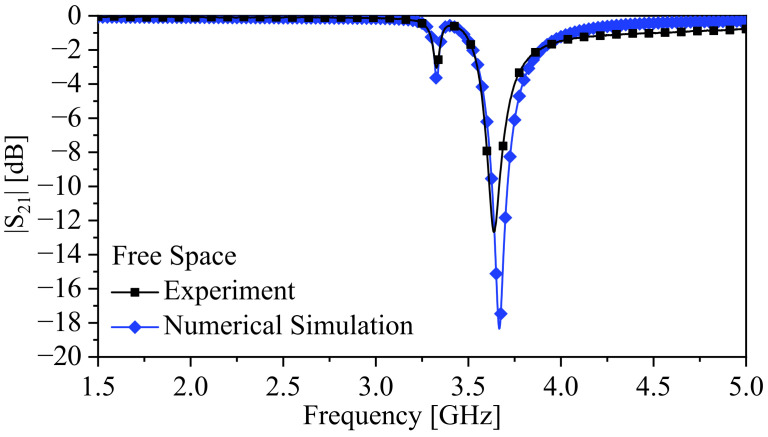
The response of the system in free space.

Figure 14 shows the response of the system in the presence of free space for both experimental and numerical results. The measured $f_{e}$ and $f_{m}$ were 3.3275 and 3.64 GHz, respectively, and using Equation (6), the calculated $\kappa_{E}$ was 0.0887, with a calculated split width of 0.3125 GHz. Comparing the split widths of the experimental and numerical results (0.338 GHz), the calculated error is 7.5 %.

**Figure 15:**
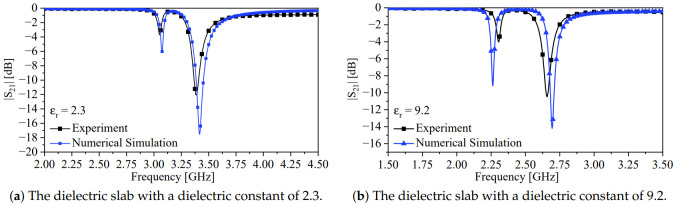
The response of the system in the presence of a dielectric slab.

Furthermore, the system was also used to detect the presence of two slabs with dielectric constants of 9.2 and 2.33. Figure 15a,b show the experimental response of the system detecting the two slabs compared with the numerical simulation. The extracted $f_{e}$ and $f_{m}$ from the response of the system detecting the slab with a dielectric constant of 2.33 were 3.055 and 3.3875 GHz, respectively, with a calculated $\kappa_{E}$ of 0.1036. Comparing the split width of the experimental (0.3325 GHz) and numerical results (0.344 GHz), the calculated error is 3.343 %. In the case of 9.2, $f_{e}$ and $f_{m}$ were 2.3025 and 2.6575 GHz, respectively, with a calculated $\kappa_{E}$ of 0.1424. Again, comparing the split widths of the experimental (0.355 GHz) and numerical results (0.431 GHz), the calculated error is 7.6%. Therefore, the results show that the loaded MUT can increase the electric coupling between the resonators, and indeed, the proposed system shows a true microwave differential sensor for dielectric materials. Table 2 summarizes the main results with calculated errors in the frequency splits between the numerical and experimental results.

Our results highlight the practical applicability of the proposed technique in utilizing coupled resonators for the design of differential sensors. This stands in contrast with other existing works, which have either explicitly or implicitly emphasized the need to completely or partially avoid the coupling between resonators [28,29,30,31,32,33]. Due to the difference in sensing mechanisms, a direct comparison of system parameters such as sensitivity levels becomes challenging to establish due to the different methodologies and approaches employed. However, Table 3 presents a general comparison with other recent related works to showcase the key features and advantages of our proposed technique.

## 5. Conclusions

In conclusion, this work has demonstrated the potential of utilizing the avoided mode splitting of coupled resonators as a novel differential sensing technique. While the study focused on planar microwave split-ring resonators, the underlying concept and methodology can be applied to other types of resonators as well. By controlling the coupling between the resonators and incorporating an MUT between them, the magnitude of the resonance frequency mode split can be controlled, allowing for the correlation between the split and the dielectric constant to be determined.

This study established the theoretical relationship between the resonators’ capacitance, the coupling capacitance, the electric coupling, and the frequency split. It was found that the system’s configuration could be further optimized to reduce the dependency of electric coupling on the resonators’ capacitance. Moreover, other types of coupling, such as magnetic and mixed coupling, can also be explored in conjunction with different resonator designs.

To validate the proposed design, a proof-of-principle experiment was conducted using fabricated resonators, and the presence of dielectric slabs with varying permittivities was detected. The experimental results were compared with 3D numerical simulations using Ansys HFSS, showing excellent agreement.

Future work in this area will focus on exploring the application of this sensing technique in different systems and domains. Potential areas of interest include biosensing, environmental monitoring, and material testing. By further refining and optimizing the proposed design, it is expected that this technique can be applied to a wide range of sensing applications, offering enhanced sensitivity and accuracy. 

## Figures and Tables

**Table 1:** Design specification of the system shown in Figure 3.

$W_{\mathrm{TL}}$ (mm)	$L_{1}$ (mm)	$L_{2}$ (mm)	$b_{1}$ (mm)	$b_{2}$ (mm)	$d_{s}$ (mm)	$L_{G}$ (mm)	$W_{G}$ (mm)	$d_{g}$ (mm)
1.56	4	11	0.2	Vari.	Vari.	50	30	0.1

**Table 2:** Summarized results.

	$f_{e}$ [GHz] (Simulation)	$f_{m}$ [GHz] (Simulation)	$f_{e}$ [GHz] (Experiment)	$f_{e}$ [GHz] (Experiment)	$f_{e}$, Error (%)	$f_{m}$, Error (%)	Width Split Error (%)
**Air**	3.667	3.329	3.64	3.3275	0.75	0.045	7.5
**$\epsilon_{r}$ = 2.3**	3.416	3.072	3.3875	3.055	0.83	0.55	3.343
**$\epsilon_{r}$ = 9.2**	2.693	2.262	2.6575	2.3025	1.32	−1.8	7.6

**Table 3:** A general comparison with other related works.

Ref.	Resonator Type	Frequency Splitting	Coupled Resonators	Breaking Symmetry	Mode Coupling Splitting
[28]	SIRs	Yes	No	Yes	No
[30]	CSRRs	Yes	No	Yes	No
[31]	SRRs	Yes	No	Yes	No
[46]	SRRs	Yes	No	Yes	No
[47]	Magnetic-LC Resonators	Yes	No	Yes	No
**This Work**	**Coupled Synchronous SRRs**	**Yes**	**Yes**	**No**	**Yes**

## Data Availability

Data generated during the study are contained within the article.

## Abbreviations

The following abbreviations are used in this manuscript:

MUT(s)	materials under test
SRR(s)	split-ring resonators
CSRR(s)	complementary split-ring resonators
SIR(s)	stepped impedance resonators
EC	electric coupling
